# Impact of the Pilates Method on Quality of Life and Functional Well-Being in Women with Osteoporosis: Protocol for a Randomized Controlled Trial

**DOI:** 10.3390/healthcare13222950

**Published:** 2025-11-17

**Authors:** Sara García-Bravo, Cristina García-Bravo, Marta Gil-Manglano, MªPilar Rodríguez-Pérez, Ana Poveda-García, Elisabet Huertas-Hoyas

**Affiliations:** 1 Department of Physical Therapy, Occupational Therapy, Physical Medicine and Rehabilitation, Rey Juan Carlos University, 28922 Alcorcón, Spain; 2 Research Group of Participation, Roles, Occupations and Activities for Community Transformation (PROACT), Rey Juan Carlos University, 28922 Alcorcón, Spain; 3 Physiocare Madrid, Physiotherapy Clinic, 28026 Madrid, Spain; 4 Research Group of Humanities and Qualitative Research in Health Science (Hum&QRinHS), Rey Juan Carlos University, 28922 Alcorcón, Spain

**Keywords:** Pilates, women, osteoporosis, quality of life, functional well-being

## Abstract

Introduction: Osteoporosis is a major public health concern among postmenopausal women, characterized by decreased bone mineral density and microarchitectural deterioration, which lead to fragility fractures, pain, functional impairment, sleep disturbances, and a reduced quality of life. Exercise, particularly strength, weight-bearing, and balance training, represents a key non-pharmacological approach to prevention and management. Pilates, a low-impact, core-centered method increasingly incorporated into rehabilitation settings, appears especially suitable for women with osteoporosis. However, high-quality randomized controlled trials concurrently evaluating its effects on pain, balance, sleep, autonomy, and health-related quality of life remain scarce. Objective: To examine the efficacy and feasibility of a Pilates-based exercise program in improving pain, balance, sleep quality, functional autonomy, and quality of life in postmenopausal women with osteoporosis. Methods: A single-blind, parallel-group, randomized controlled trial will be conducted over 12 weeks at Physiocare Madrid (Spain). A total of 126 (63 per group) postmenopausal women aged 50–80 years, diagnosed with osteoporosis by densitometry or with a prior fragility fracture, will be randomly assigned (1:1; OxMaR software, version 2014) to one of two groups: (a) Experimental group: supervised Pilates mat sessions, 60 min, twice weekly for 12 weeks; or (b) Control group: ergonomics education for activities of daily living, two 60 min sessions held six weeks apart. Outcome assessors will remain blinded to group allocation. Evaluations will be conducted at baseline and post-intervention. Outcome measures will include balance and mobility (Timed Up and Go Test; Functional Reach Test), functional autonomy (Functional Independence Measure), pain intensity (Visual Analog Scale), sleep quality (Pittsburgh Sleep Quality Index), health-related quality of life (WHOQOL-BREF; QUALEFFO-41), and treatment satisfaction (CSQ-8). Feasibility parameters (recruitment, adherence, retention, and safety) will also be monitored. Data will be pseudonymized and analyzed descriptively to estimate variability and preliminary effects, informing the design of a definitive trial. Expected Results: It is hypothesized that Pilates will produce clinically meaningful improvements in balance, pain, sleep quality, and health-related quality of life compared with ergonomics education, with acceptable feasibility and safety outcomes. Conclusions: This randomized controlled trial will provide initial evidence regarding the efficacy and feasibility of Pilates as a complementary rehabilitation strategy for women with osteoporosis and provide key parameters to optimizing a future adequately powered trial. Ethics and Dissemination: This study will be conducted in accordance with the principles of the Declaration of Helsinki and has been approved by the Human Ethics Committee of Universidad Rey Juan Carlos. Potential risks will be minimized, and any adverse events will be systematically recorded and addressed.

## 1. Introduction

Osteoporosis is a systemic skeletal disorder characterized by reduced bone mineral density and deterioration of bone microarchitecture, resulting in increased bone fragility and a heightened risk of fractures even after minimal trauma. It constitutes a major global public health issue, particularly affecting postmenopausal women. Worldwide, approximately 200 million women are estimated to suffer from osteoporosis, with prevalence rising sharply with age. Nearly one in three women over 50 years of age will experience at least one fragility fracture during their lifetime [1]. In Spain, recent data indicate that approximately 18.6% of women over 50 years of age have osteoporosis, compared with 2.6% of men, and this figure increases to nearly 25% among women aged 65 years or older [2].

A particularly concerning feature of osteoporosis is its “silent” nature. Bone loss progresses asymptomatically until a fracture occurs, leading to frequent underdiagnosis and undertreatment [2]. Many patients remain unaware of their personal risk or fail to recognize the condition until after their first fragility fracture, causing delays in diagnosis and management [1]. Consequently, a substantial proportion of women do not receive timely preventive or therapeutic interventions [3].

The most direct clinical consequence of osteoporosis is the occurrence of fragility fractures, most commonly affecting the hip, vertebral column, wrist, and shoulder. These fractures cause acute pain and may lead to severe complications. Collectively, osteoporotic fractures have a profound impact on patients’ health, resulting in chronic pain, reduced quality of life, and increased hospitalization and disability rates [3,4]. Among these, hip fractures are of particular concern due to their high morbidity and mortality within the first year following the event, as well as the frequent loss of functional independence. Many patients are unable to regain their previous ability to walk or live independently.

Vertebral fractures may also lead to structural deformities such as thoracic kyphosis (osteoporotic hump) and postural alterations that disrupt body biomechanics. These deformities are often associated with chronic back pain and restricted trunk mobility [5,6]. Even in the absence of new fractures, established osteoporosis may cause trabecular microfractures—especially in vertebrae—that produce persistent pain and stiffness, further limiting physical activity [3,4,5,6]. Consequently, osteoporosis contributes to significant functional impairments, including decreased muscle strength, impaired balance, and reduced ability to perform daily activities, thereby increasing dependence on others for basic self-care [4,6]. Several studies have demonstrated that the decline in physical function and mobility associated with osteoporosis is comparable to that observed in other disabling chronic diseases [7].

Another critical aspect of osteoporosis is the increased risk of falls. Bone fragility heightens the likelihood of sustaining fractures after minimal trauma; thus, even a fall from standing height can result in serious injury. Among older adults in the general population, approximately 30–35% experience at least one fall each year, with a higher incidence in women due to factors such as sarcopenia and age-related impairments in balance and postural control [4,6]. In individuals with osteoporosis, these falls represent the leading cause of fragility fractures. This situation creates a vicious cycle: age-related declines in muscle mass and strength, coupled with neuromuscular deterioration, predispose individuals to falls; in the presence of fragile osteoporotic bone, such falls are more likely to cause fractures, which in turn exacerbate immobility and muscular weakness.

Beyond its physical consequences, osteoporosis exerts profound psychosocial and quality-of-life impacts. Functional limitations and chronic pain resulting from fractures, or from bone fragility itself, can negatively influence mood, confidence, and self-esteem. Even in the absence of fractures, many women with osteoporosis experience a persistent fear of falling or sustaining injury—often referred to as kinesiophobia or fear of movement—which leads to self-imposed reductions in physical activity [7,8]. Paradoxically, this sedentary behavior accelerates muscle loss and worsens balance impairments, further increasing the risk of falls.

Chronic pain also interferes with emotional well-being. Persistent discomfort and functional restrictions are often associated with anxiety and depressive symptoms. Consequently, osteoporosis not only causes pain and limits mobility but also contributes to social isolation and diminished self-esteem [4,6,7,8]. A study conducted among postmenopausal women with osteoporosis but without prior fractures reported that 58% presented symptoms of depression and 55% experienced chronic pain, along with significantly lower scores for quality of life and self-esteem compared with women without osteoporosis [9,10]. These findings highlight that the psychological burden of osteoporosis may arise even before fractures occur, substantially affecting emotional well-being and overall quality of life [4,9].

It is also important to emphasize the complex interplay between pain, sleep disturbances, and mood disorders in women with osteoporosis. Chronic osteoporotic pain—such as low back pain caused by vertebral compression fractures—often disrupts sleep, leading to insomnia or non-restorative sleep patterns. In turn, sleep deprivation heightens pain perception and contributes to daytime fatigue and irritability. Reciprocal relationships among poor sleep quality, pain, and depressive symptoms have been consistently observed in postmenopausal women with osteoporosis, where each factor exacerbates the others in a self-reinforcing cycle [4,5,6].

Sleep disturbances not only impair quality of life but may also increase the risk of falls due to drowsiness, reduced attention, and impaired gait stability. Consequently, osteoporosis involves multidimensional clinical consequences—physical (fractures, pain, disability), psychological (fear, depression, anxiety), and social (isolation, dependence)—that collectively impair health-related quality of life in affected women [4,5,6].

The management of osteoporosis generally includes pharmacological treatment with antiresorptive and anabolic agents, complemented by calcium and vitamin D supplementation [4]. Nevertheless, physical exercise is widely recognized as a cornerstone of both prevention and rehabilitation. Regular exercise contributes to attenuating bone loss, enhancing muscular strength, and improving postural control [8,10,11]. In particular, strength training, weight-bearing activities, and balance exercises are strongly recommended to reduce the risk of falls and promote greater functional independence [8,10,11].

The Pilates Method, developed by Joseph Pilates in the 1920s, focuses on achieving precise control of body position and movement [12]. It consists of a series of mind–body exercises designed to strengthen, stabilize, and increase flexibility of the body’s center or “core,” while emphasizing muscular control, postural alignment, and conscious breathing [13]. The method encompasses 33 exercises structured around seven fundamental principles: concentration, breathing, control, flow of movement, centering, and precision [12,13].

Although initially conceived as a form of physical exercise, the Pilates Method has increasingly been incorporated into rehabilitation programs aimed at reducing pain and disability, particularly in patients with musculoskeletal and rheumatologic conditions [14,15]. Its low-impact nature makes it especially suitable for women with osteoporosis, as it minimizes high-risk movements such as deep spinal flexion. Preliminary studies have demonstrated that Pilates can improve muscle strength, balance, pain, and overall quality of life in postmenopausal women with osteoporosis [11,16,17]. Furthermore, additional benefits have been observed in sleep quality, anxiety, and depressive symptoms [9].

Despite these promising findings, the current body of evidence remains limited. Most existing studies are constrained by small sample sizes, short intervention periods, and methodological heterogeneity [11,17]. To date, few high-quality randomized controlled trials have simultaneously assessed multiple clinically relevant outcomes, such as quality of life, pain, balance, sleep, and functional performance. Therefore, there is a clear need for a well-designed randomized controlled trial (RCT) to comprehensively evaluate the efficacy and feasibility of the Pilates Method in women with osteoporosis.

## 2. Materials and Methods

### 2.1. Study Design

A single-blind, RCT with a three-month intervention period will be conducted at Physiocare Madrid, located in the Autonomous Community of Madrid, Spain.

The development of this study protocol followed the SPIRIT 2025 guidelines [18]. This study will be prospectively registered at ClinicalTrials.gov and conducted in accordance with the CONSORT 2010 guidelines to ensure transparent and accurate reporting of its methodology and outcomes [19].

### 2.2. Sample Description

Participants receiving treatment at Physiocare Madrid will be invited to participate in this study. Recruitment will be conducted through informational posters displayed in various areas of the center and through an informational session specifically addressed to potential participants.

The inclusion criteria will be postmenopausal women (≥12 months of amenorrhea) aged 50–80 years; a documented diagnosis of osteoporosis confirmed by bone densitometry (T-score ≤ −2.5 at the lumbar spine, total hip, or femoral neck), or a history of medically or radiologically confirmed fragility fracture; absence of associated metabolic disorders such as Crohn’s disease; availability to attend two sessions per week for 12 consecutive weeks; and voluntary participation with the provision of written informed consent.

The exclusion criteria will include any medical contraindication to physical exercise; current participation in another similar intervention program during the same period; or refusal to participate voluntarily and/or failure to provide written informed consent.

Pharmacological treatment related to osteoporosis (including bisphosphonates, denosumab, teriparatide, or calcium and vitamin D supplementation) will not constitute an exclusion criterion, provided that the treatment has remained stable for at least three months prior to enrollment. All pharmacological treatments will be recorded as part of each participant’s clinical profile and considered as potential confounding variables during statistical analysis.

### 2.3. Procedure

Individuals meeting the inclusion criteria will be invited to voluntarily participate in this study. They will receive detailed information about the intervention, study procedures, potential risks, and expected benefits, enabling them to make an informed decision regarding participation. Those who agree to take part will be required to sign a written informed consent form (see Supplementary File S1).

Following the provision of informed consent, sociodemographic data will be collected, including name, age, sex, and any ongoing conventional therapies. Upon enrollment, each participant will be pseudonymized through the assignment of a unique alphanumeric identifier composed of the letter “P” followed by a sequential number corresponding to the order of inclusion (e.g., P01, P02, P03, etc.). These codes will be used in all study documentation and databases to ensure confidentiality and protect participant identity. The correspondence between the assigned codes and personal identifiers will be recorded in a secure file accessible exclusively to the principal investigator (PI).

All personal data obtained through informed consent will be processed in compliance with the European Union General Data Protection Regulation (EU Regulation 679/2016). Data will be stored in a secure research database accessible only to the study investigators.

All data collected during baseline and follow-up assessments will be entered into a Microsoft Excel database and subsequently exported to IBM SPSS Statistics (version 27) for analysis. Descriptive statistics (means, standard deviations, and frequency distributions) will be computed to characterize the sample and summarize the main study variables. Inferential analyses will be conducted to examine between-group and within-subject differences over time, using repeated-measures ANOVA or ANCOVA when appropriate, with baseline values included as covariates. Effect sizes and 95% confidence intervals will be reported to estimate the magnitude and precision of the observed effects. All statistical tests will be two-tailed, with the significance level set at *p* < 0.05. Missing data will be handled according to the intention-to-treat (ITT) principle, applying multiple imputation techniques when necessary to maintain statistical power and minimize bias. Access to pseudonymized datasets will be restricted to the research team, whereas access to identifiable personal information will be limited exclusively to the PI.

The sample size was estimated using G*Power version 3.1, based on a two-tailed independent samples *t*-test comparing the means between two groups (experimental and control). The following parameters were used: an effect size (Cohen’s d) of 0.58, a significance level (α) of 0.05, a statistical power (1−β) of 0.85, and an allocation ratio (N_2_/N_1_) of 1. Under these assumptions, the required sample size was 110 participants in total (55 per group) to detect a moderate effect size with 85% power and a 5% type I error rate. To account for potential attrition of 15% during the 12-week follow-up, the final recruitment target was increased to approximately 126 participants in total (63 per group).

### 2.4. Intervention

This study will employ a RCT design with parallel groups and a single-blind approach (evaluator blinding).

Participant allocation will be performed using computer-generated randomization. The randomization sequence will be created with the OxMaR software, a tool specifically designed for minimization and randomization in clinical trials [20]. This software will enable the random assignment of participants into two groups: a control group (CG), which will receive education on postural ergonomics techniques applied to activities of daily living, and an experimental group (EG), which will participate in Pilates Method sessions.

Each participant’s allocation number will be sealed in an opaque envelope and delivered to the researchers responsible for implementing the interventions in both the CG and EG. The evaluator conducting the outcome assessments will remain blinded to group allocation and will not have access to any information regarding participants’ assigned groups.

Following randomization and baseline assessment, the 12-week intervention period will begin. Upon completion, the same assessment battery will be re-administered to evaluate post-intervention outcomes and determine whether statistically significant differences exist between groups.

The CG will participate in two educational sessions on ergonomics and postural hygiene, each lasting 60 min and held six weeks apart. These sessions will aim to improve functional performance during activities of daily living by promoting safe movement strategies, spinal alignment, proper body mechanics, and fall prevention. This approach was designed in accordance with current clinical practice guidelines and expert recommendations on ergonomics and postural hygiene in osteoporosis management [21,22,23]. Each session will be jointly conducted by a physiotherapist and an occupational therapist with experience in osteoporosis management. Participants will receive illustrated brochures and short video demonstrations developed by the research team, based on established postural hygiene guidelines, to reinforce learning.

The EG will undergo Pilates Method training consisting of one-hour sessions held twice weekly for 12 consecutive weeks. All Pilates exercises will be performed on a mat and will emphasize the core activation principles characteristic of the method. Sessions will be supervised by a physiotherapist and an occupational therapist certified in the Pilates Method, both with over ten years of professional experience in the management of individuals with osteoporosis and musculoskeletal conditions. The specific intervention protocol for the EG is detailed in Table 1.

**Figure 1:**
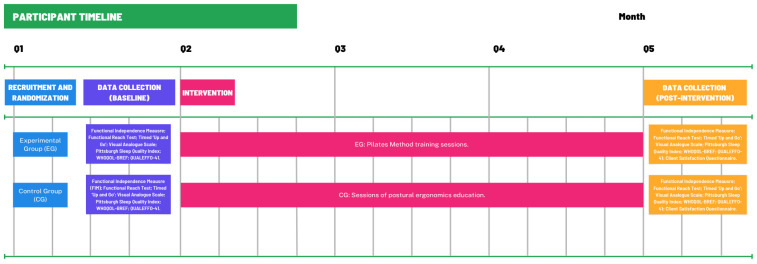
Participant Timeline.

An individualized baseline assessment will be performed to adapt the exercises to each participant’s physical condition and functional limitations, ensuring that all contraindicated movements are avoided. The training intensity will be monitored using the Borg CR-10 [24] perceived exertion scale, maintaining a moderate intensity level between 4 and 6 points, corresponding to approximately 50–70% of the maximum heart rate (%HRmax). Participants will wear smart bands (Xiaomi Smart Band 9 Active) during all sessions to continuously record heart rate and oxygen saturation, ensuring objective consistency in workload monitoring. Each participant will be informed of their individual %HRmax prior to starting the sessions to facilitate self-awareness and control of exercise intensity. Each session will include approximately 20 min of warm-up, 30 min of core Pilates exercises, and 10 min of cool-down, with short rest intervals of 30–60 s between exercises included within the total session duration. The number of repetitions and weekly progression of the exercises are detailed in Table 1. Continuous supervision will be provided throughout all sessions to guarantee a safe and controlled training environment. In the event of any discomfort or adverse incident, the activity will be immediately discontinued. The participant timeline is illustrated in Figure 1.

### 2.5. Measures

An initial assessment will be conducted during which the evaluation tests described below will be administered. A second assessment will take place following the intervention period (after two months).

#### 2.5.1. General Medical Information

Data on participants’ general health status will be collected from the documentation provided by each individual and will include information on sex, age, medical and social history, and the number of weekly hours of occupational therapy and physiotherapy received.

#### 2.5.2. Functional Capacity Assessment

- Functional Independence Measure (FIM) [25]: This instrument assesses the degree of functional independence in activities of daily living, providing information on both motor and cognitive performance through 18 items. Each item is scored on a 7-point ordinal scale (1–7), with higher scores indicating greater levels of independence. The assessment is conducted through direct observation and interview. The FIM has shown excellent inter-rater reliability (ICC > 0.90) and internal consistency, and the Spanish version has been validated for clinical and research use [25,26].
- Timed “Up and Go” (TUG) [27]: This test measures, in seconds, the time it takes for an individual to stand up from a chair, walk three meters, turn around, return to the chair, and sit down again. It serves as an indicator of balance and gait speed, and functional mobility. The TUG has demonstrated high reliability (ICC = 0.95) and validity for predicting fall risk in older adults, and its Spanish adaptation has been validated in geriatric and musculoskeletal populations [28].
- Functional Reach Test (FRT) [29]: This test evaluates the maximum distance an individual can displace their center of gravity toward the limits of their base of support without losing balance. Standing upright with feet hip-width apart, the participant flexes the shoulder to 90°, extends the arm forward, and reaches as far as possible. The examiner records the distance reached. Two practice trials and three recorded trials are performed, and the mean distance (in centimeters) is used for analysis. The FRT has excellent test–retest reliability (ICC > 0.80) and validity for assessing dynamic balance [30].
- Visual Analog Scale (VAS) [31]: This self-reported scale consists of a 10 cm horizontal line representing perceived pain intensity, anchored by “no pain” (0) and “worst imaginable pain” (10). Participants mark the point that best represents their current pain level. The resulting score (0–10) corresponds to the marked value, with higher scores indicating greater pain intensity. The VAS is a simple, sensitive, and validated tool for assessing pain in adults with musculoskeletal disorders, including osteoporosis.
- Pittsburgh Sleep Quality Index (PSQI) [32,33]: This self-administered questionnaire includes 19 items evaluating sleep quality over the previous month. The items are grouped into seven components: subjective sleep quality, sleep latency, sleep duration, habitual sleep efficiency, sleep disturbances, use of sleep medication, and daytime dysfunction. Each component is scored from 0 to 3, generating a global score ranging from 0 to 21, with higher scores indicating poorer sleep quality. A total score greater than 5 is generally considered indicative of poor sleep. The Spanish version of the PSQI has demonstrated adequate reliability and validity and is considered an appropriate tool for assessing sleep quality in adults [32,33].

#### 2.5.3. Treatment Satisfaction and Quality-of-Life Assessment

- Client Satisfaction Questionnaire (CSQ-8) [34]: This self-administered questionnaire consists of eight items that assess participants’ satisfaction with the care received, the perceived quality of services, and the extent to which their expectations were met. Each item is scored on a 4-point Likert scale, yielding a total score of up to 32 points, with higher values indicating greater satisfaction. The Spanish adaptation preserves the psychometric properties of the original version and is considered suitable for evaluating satisfaction with health services in Spanish-speaking populations. The CSQ-8 will be administered to both treatment groups [35].
- WHOQOL-BREF [36]: This self-administered questionnaire assesses an individual’s perceived overall quality of life and general health. It comprises four domains: physical health, psychological health, social relationships, and environment. Higher scores reflect better quality of life. The Spanish validation of the WHOQOL-BREF has demonstrated strong internal consistency (α = 0.87) and cross-cultural reliability [37].
- Quality of Life Questionnaire of the European Foundation for Osteoporosis (QUALEFFO-41) [38]: This self-administered questionnaire includes 41 items designed to measure health-related quality of life in individuals with osteoporosis, particularly those with vertebral fractures. It evaluates five domains: pain, physical function, social function, general health perception, and mental well-being. Items are rated using a Likert-type scale, and the resulting scores are transformed to a 0–100 scale, where higher values indicate poorer quality of life. The Spanish version has shown good internal consistency (α = 0.88) and construct validity for use in osteoporotic populations [39].

Several strategies will be implemented to promote participant retention and ensure complete follow-up, including regular communication, flexible scheduling of sessions, and individualized monitoring to maintain engagement. In cases where participants discontinue or deviate from the intervention protocol, efforts will be made to collect all available outcome data up to the point of withdrawal, including post-intervention assessments when feasible, to preserve data integrity and enable intention-to-treat analyses.

A Data Monitoring Committee (DMC) was not established for this study. Given its single-center design, non-pharmacological nature, and the minimal risks associated with the intervention, an independent DMC was not deemed necessary. Participant safety and adherence to the study protocol will be continuously monitored by the principal investigator and the research team. This decision aligns with the SPIRIT 2013 and ICH-GCP (E6 R2) guidelines, which recommend DMC oversight primarily for multicenter or high-risk clinical trials [40]. Any adverse events or protocol deviations will be documented and reported to the Research Ethics Committee of Universidad Rey Juan Carlos in accordance with institutional and ethical requirements.

## 3. Ethics and Dissemination

### 3.1. Ethics

This project was approved by the Research Ethics Committee of Universidad Rey Juan Carlos prior to its initiation (in October 2025 with the approval code 141020256932025). This study was conducted in accordance with the ethical principles outlined in the Declaration of Helsinki for medical research involving human subjects, originally adopted at the 18th World Medical Association (WMA) Assembly (Helsinki, Finland, June 1964) and last revised at the 64th WMA General Assembly (Fortaleza, Brazil, October 2013). Furthermore, this study complies with current national regulations, including Law 14/2007 on Biomedical Research and Royal Decree 223/2004.

### 3.2. Data Processing

Participant data will be recorded in a secure digital file containing each participant’s medical history and completed assessments, identified exclusively by a unique code accessible only to the PI.

The collaborating institution involved in this study has signed a formal collaboration agreement provided by the PI and has accepted full responsibility for maintaining data confidentiality.

Upon enrollment, all participants will be pseudonymized through the assignment of a unique alphanumeric identifier consisting of the letter “P” followed by a sequential number corresponding to the order of inclusion (e.g., P01, P02, P03, etc.). These identifiers will be used in all study documents and databases to ensure confidentiality and protect participant identity.

The correspondence between the assigned codes and personal identifiers will be stored in a master list, kept in a secure location accessible solely to the principal investigator.

### 3.3. Potential Risks and Benefits

Participation in this study involves minimal risk, comparable to that associated with any moderate-intensity physical exercise program. Potential adverse effects may include temporary muscle soreness or fatigue.

To minimize these risks, all sessions will be conducted by a physiotherapist and an occupational therapist certified in the Pilates Method, both with experience in its application for individuals with osteoporosis. An individualized baseline assessment will be carried out to tailor the exercises to each participant’s physical abilities and limitations, avoiding contraindicated movements. In addition, continuous supervision will be provided throughout all sessions to ensure a safe and controlled environment. In the event of any discomfort or adverse incident, the activity will be immediately discontinued and reported to the research team.

Participation in this study may lead to improvements in muscle strength, balance, posture, and mobility, factors that contribute to the prevention of falls and fractures. Moreover, regular practice of the Pilates Method may enhance perceived well-being, sleep quality, and overall health-related quality of life.

Although direct individual benefit cannot be guaranteed, the results of this study are expected to provide valuable scientific evidence regarding the effectiveness of the Pilates Method as a complementary rehabilitation approach for women with osteoporosis, thereby contributing to the advancement of safe and effective rehabilitation strategies.

## 4. Discussion

This protocol addresses an important clinical need arising from the scarcity of comprehensive non-pharmacological physical interventions targeting function, quality of life, pain, sleep, balance, and treatment satisfaction in postmenopausal women with osteoporosis. To this end, validated assessment tools, including the FIM, TUG, Functional Reach Test, VAS, PSQI, WHOQOL-BREF, QUALEFFO-41, and CSQ-8, will be employed.

Osteoporosis, due to its multidimensional nature, represents a complex condition that encompasses not only bone loss and fragility fractures but also chronic pain, fear of falling, anxiety and depressive symptoms, and limitations in daily activities. Although pharmacological treatments have proven effective in reducing fracture risk, the associated psychosocial deterioration often compromises patients’ autonomy and quality of life [2,3,4,6].

In this context, the Pilates Method emerges as a promising approach to address these multifaceted challenges, emphasizing postural control, core activation, breathing, and low-impact movement [12,13,14]. Pilates may exert its beneficial effects through multiple mechanisms.

First, it improves balance and postural control by activating trunk-stabilizing muscles and promoting proper body alignment, potentially enhancing performance in balance-related tests such as the Functional Reach Test and TUG [11,17]. Second, it strengthens muscles and increases endurance, leading to improved motor performance and functional independence [3,4]. Moreover, Pilates may help reduce chronic pain by improving postural alignment, redistributing mechanical loads, and incorporating breathing and body-awareness techniques [9,11,16]. It has also been associated with better sleep quality and psychological well-being, as supervised low-impact exercise can reduce anxiety and fear of falling [5,8]. Notably, Aibar-Almazán et al. [9] reported that a Pilates program improved sleep quality, depression, and anxiety in postmenopausal women. Furthermore, reductions in kinesiophobia, pain, and functional limitations have been observed following Pilates interventions, reinforcing participants’ perception of safety in movement and their overall quality of life, thereby supporting Pilates as a safe and effective form of exercise [16].

Although this protocol does not specifically target bone mineral density (BMD), previous studies have demonstrated improvements in several of the variables analyzed in the present research. Cronin et al. [15] and McLaughlin et al. [17] reported no significant changes in lumbar spine or hip BMD but did observe improvements in functional capacity and physical well-being. Similarly, Fernández-Rodríguez et al. [11] found that, even in the absence of BMD changes, participants achieved clinically meaningful improvements in function and quality of life. Therefore, this study prioritizes functional, pain-related, and quality-of-life outcomes, aligning with the current research trend emphasizing functional recovery over bone density alone.

Beyond osteoporosis, a growing body of evidence supports the efficacy of the Pilates Method across various chronic conditions. García-Bravo et al. [41] observed significant improvements in balance, coordination, gross and fine motor dexterity, self-perception, and independence in activities of daily living among individuals’ post-stroke. Çoban et al. [42] reported improvements in dynamic balance in patients with Parkinson’s disease following Pilates training. Likewise, in individuals with multiple sclerosis, Pilates has been associated with enhanced gait, balance, fatigue, strength, stability, and health-related quality of life [43,44].

A potential limitation of this study is the imbalance in the amount of contact time between the intervention and control groups. While the experimental group will participate in 24 supervised Pilates sessions, the control group will only attend two educational sessions on ergonomics and daily living activities. This difference in exposure and interaction with healthcare professionals may introduce an attention or expectation bias, potentially influencing participants’ perception of improvement or adherence. However, this design choice was made to ensure that the control group receives a minimal but ethically appropriate level of health education, without introducing confounding physical activity components. The potential impact of this imbalance will be acknowledged and carefully considered when interpreting the study results.

## 5. Conclusions

This protocol represents a valuable contribution to the field of rehabilitation, as it outlines a RCT designed to evaluate the efficacy of the Pilates Method in improving pain, balance, sleep quality, autonomy, and quality of life in women with osteoporosis. The findings of this study may provide a foundation for future research aimed at further establishing the Pilates Method as a safe and effective complementary intervention to enhance functional capacity, independence, and overall well-being in this population.

## Figures and Tables

**Table 1:** Pilates intervention protocol for EG.

	Phase	Exercise (Number of Repetitions)	Time
Week 1, 5 and 9	Warm-up	Breathing (15)	5 min
		Head nods (8)	5 min
		Imprint and release (10)	5 min
		Hip release (8)	5 min
	Main exercise	Leg slides (8)	5 min
		Hip Rolls (8)	5 min
		Single leg stretch (8)	5 min
		One leg circle (10)	5 min
		Shoulder bridge (8)	5 min
		Swimming prep (5)	5 min
	Cool-down	Breathing and relaxation (5)	2 min
		Spine stretch forward (4)	1 min
		Shell stretch (8)	2 min
		Cat stretch (6)	2 min
		Static muscle stretching (6)	2 min
		Gentle pelvic tilts (4)	1 min
Week 2, 6 and 10	Warm-up	Breathing (15)	5 min
		Head nods (8)	5 min
		Arm circles (8)	5 min
		Pelvic clock (10)	5 min
	Main exercise	Swimming prep (5)	5 min
		Side leg lift series (lift and lower, top leg circles, staggered legs and both legs together) (4)	15 min
		One leg balance (6)	5 min
	Cool-down	Breathing and relaxation (5)	2 min
		Spine stretch forward (8)	2 min
		Half roll back (6)	2 min
		Cat stretch (6)	2 min
		Static muscle stretching (6)	2 min
Week 3, 7 and 11	Warm-up	Breathing (15)	5 min
		Shoulder rolls (8)	5 min
		Hip release (8)	5 min
		Clam Shell (10)	5 min
	Main exercise	Hundred (Adapted) (1)	5 min
		Hip Rolls (8)	5 min
		Hip twist (8)	5 min
		Dead bug (6)	5 min
		Scissors on the mat (8)	5 min
		Leg pull front (6)	5 min
	Cool-down	Breathing and relaxation (5)	1 min
		Shoulder opener (8)	2 min
		Spine stretch (axial) (8)	2 min
		Static muscle stretching (10)	5 min
Week 4, 8 and 12	Warm-up	Breathing (15)	5 min
		Arm openings (8)	5 min
		Hip Rolls (8)	5 min
		Leg slides (8)	5 min
	Main exercise	Squats (10)	5 min
		Side kick kneeling (8)	5 min
		Side bend (8)	5 min
		Side leg lift standing (6)	5 min
		Leg kick on unstable platform (8)	10 min
	Cool-down	Breathing and relaxation (5)	1 min
		Cat stretch (4)	1 min
		Static muscle stretching (20)	8 min

All exercises will be individually adapted to the participants’ abilities and characteristics.

## Data Availability

The original contributions presented in this study are included in the article and Supplementary Materials. Further inquiries can be directed to the corresponding author.

## Supplementary Materials

The following supporting information can be downloaded at: https://www.mdpi.com/article/10.3390/healthcare13222950/s1, File S1: Consent Form.
