# Supplementary material for: Impact of the Pilates Method on Quality of Life and Functional Well-Being in Women with Osteoporosis: Protocol for a Randomized Controlled Trial
Source: Healthcare (Basel). 2025 Nov 17;13(22):2950. doi: 10.3390/healthcare13222950 (PMC12652029; doi:10.3390/healthcare13222950)
Supplement: Supplementary file 1 [file healthcare-13-02950-s001.zip › healthcare-3976891-supplementary.pdf]

Universidad
Rey Juan Carlos

INFORMATION SHEET AND INFORMED CONSENT FORM FOR PARTICIPANTS

STUDY/PROJECT TITLE: Effectiveness of Rehabilitation Through the Pilates Method in Women with Osteoporosis.

PRINCIPAL INVESTIGATOR: Cristina García Bravo

Thank you for your interest in participating in this study. Before you decide whether to take part, it is important that you understand the purpose of the research, what your participation will involve, and what potential benefits and risks may be associated with it.

Please take as much time as you need to read the following information carefully, and feel free to ask any questions you may have to the researcher whose contact details appear above, so that you can make an informed decision.

Please note that your participation in this study is entirely voluntary. You are free to decline to participate or to withdraw your consent at any time without providing a reason and without any consequences to you.

What is this study and what is its purpose?

This study aims to explore the effectiveness of the Pilates Method in improving pain, balance, sleep quality, autonomy, and overall quality of life in women with osteoporosis.

The need for this research arises from the interest in exploring and promoting new intervention approaches for women with osteoporosis, beyond conventional treatments.

The purpose of this study is to evaluate the effects of the Pilates Method as a complement to usual care, in order to determine whether this form of exercise can help improve quality of life, reduce pain, enhance balance, promote better sleep, and increase functional ability in daily activities.

It is expected that participants may benefit from new forms of physical and occupational therapy that enhance their well-being and autonomy. Furthermore, the results of this study may serve to inform and guide healthcare professionals on alternative interventions aimed at improving well-being in women with osteoporosis.

Who is conducting this study?

The principal investigator is Cristina García-Bravo, Occupational Therapist and Professor at Rey Juan Carlos University.

You may contact her by phone at +34 914884877 or by email at cristina.bravo@urjc.es. Please do not hesitate to reach out if you have any questions or require further clarification.

The evaluations and the intervention being studied will be carried out by Marta Gil Manglano, Occupational Therapist at Physiocare Madrid, Elisabet Huertas-Hoyas, MªPilar Rodríguez-Pérez, Ana Poveda-García and Sara García Bravo, Professor at Rey Juan Carlos University, together with the principal investigator.

How long will the study last?

The expected duration of this study is three months. First, a physical assessment of your current condition will be conducted. Afterwards, you will be randomly assigned to one of two groups: the experimental group or the control group.

If you are assigned to the experimental group, you will take part in a 12-week intervention consisting of two 60-minute sessions per week. A follow-up assessment will be carried out at the end of the program.

If you are assigned to the control group, you will participate in a 12-week intervention consisting of two 60-minute sessions, one session every six weeks, followed by a final assessment at the end of the study.

You will not need to travel to any additional location, as all sessions and evaluations will take place at Physiocare Madrid..

How will the study be conducted? What will my participation involve and what are the potential risks?

The study will be conducted at the Physiocare Madrid facilities, specifically in the center's gymnasium.

You will complete eight assessment tests before and after the study, during the first and last week of the intervention.

The assessment tools will include questionnaires and observations evaluating your performance, pain, quality of life, sleep quality, and balance.

You will be asked to actively participate in the sessions, following the schedule established for this study over the course of 12 weeks. The intervention will take place from Monday to Friday, and your participation will not be required on weekends.

Please note that family members will not be allowed to attend the intervention sessions.

Participation in this study involves minimal risk, similar to that associated with any moderate-intensity physical exercise program. Possible adverse effects may include temporary muscle discomfort or fatigue.

To minimize these risks, all sessions will be conducted by a certified occupational therapist trained in the Pilates Method, with experience working with individuals with osteoporosis. An

initial individual assessment will be performed to tailor the exercises to each participant's abilities and limitations, avoiding any contraindicated movements. In addition, continuous supervision will be maintained throughout all sessions to ensure a safe and controlled environment.

In the event of any discomfort or incident, the activity will be immediately suspended, and the research team will be notified.

What data will be collected for this study?

The following data will be collected for the purposes of this study:

- Name, age, sex, and number of treatment hours received per week.
- Scores obtained in the assessment scales administered before and after the study.
- Personal signature (for the informed consent form).

How is confidentiality and personal data protection ensured?

This study involves the processing of personal data. The researchers will guarantee the confidentiality of all information collected, strictly complying with data protection regulations, in particular Regulation (EU) 2016/679 of 27 April (General Data Protection Regulation – GDPR), as well as Spanish Organic Law 3/2018 of 5 December on the Protection of Personal Data and the Guarantee of Digital Rights.

In accordance with data protection legislation, we inform you of the following:

After participants are enrolled in the study, their data will be pseudonymized through the assignment of a unique alphanumeric code. This code will consist of the letter "P" followed by a sequential number corresponding to the order of inclusion in the study (for example: P01, P02, P03, etc.).

These codes will be used in all study documents and databases to ensure confidentiality and protect participant identity. The correspondence between the assigned codes and personal data will be recorded in a master list, which will be securely stored and accessible only to the principal investigator.

DATA CONTROLLER

The data controller is Rey Juan Carlos University (Universidad Rey Juan Carlos), located at C/ Tulipán s/n, 28233 Móstoles, Madrid, Spain.

CONSENT AND PURPOSE

Your personal data will be processed with your explicit consent, within the framework of the research activities conducted at Rey Juan Carlos University. The data will be used solely for the purposes of the present study and may only be used for additional and compatible research purposes after the data have been fully anonymized.

You may withdraw your consent at any time, without providing a reason and without any consequences for you.

DATA DISCLOSURE

The personal data collected will not be shared or transferred without your explicit consent, except in cases where there is a legal obligation to do so, or after pseudonymization, when re-identification would only be possible for the purpose of notifying participants about the results related to their own intervention.

DATA RETENTION

Your personal data will be retained only for the duration of the project, after which they will be securely destroyed.

EXERCISE OF RIGHTS

In accordance with your right to the protection of personal data, you are informed that you may exercise your rights of access, rectification, erasure, restriction of processing, objection, and any other rights recognized under the General Data Protection Regulation (GDPR) and Spanish Organic Law 3/2018 on the Protection of Personal Data and the Guarantee of Digital Rights at any time.

You may exercise these rights by submitting a request to Rey Juan Carlos University, C/ Tulipán s/n, 28933 Móstoles (Madrid), through the university's official registry, its electronic headquarters, or by email at protecciondedatos@urjc.es.

You may also request further information or clarification regarding the exercise of your rights by contacting the Data Protection Officer (DPO) of Rey Juan Carlos University at protecciondedatos@urjc.es.

If you believe that your rights have not been fully respected, you may lodge a complaint with the Spanish Data Protection Agency (Agencia Española de Protección de Datos) at C/ Jorge Juan, 6 – 28001 Madrid, or through its website at www.aepd.es.

Further information regarding the protection of your personal data can be found at: https://www.urjc.es/proteccion-de-datos.

END OF INFORMATION SHEET

This document concludes the information section for you to consider whether or not you wish to participate in the study.

Please remember that you are encouraged to ask any questions or request any clarification you may need to ensure that you have all the necessary information before making your decision.

If you decide to participate, we kindly ask you to complete and sign the following page, titled "Informed Consent", indicating that you agree to take part in the study after having received and understood all the relevant information.

INFORMED CONSENT FORM

I, (name of participant/patient or their representative):

- On my own behalf (check if applicable)
- On behalf of another person (check if applicable).

  Name of the person I represent: ________________________________

Acknowledging that I have taken into account their previously expressed wishes or objections regarding this study,

confirm that I have read the information sheet provided to me. I declare that I have understood its contents and that I have been given the opportunity to ask any questions I considered necessary to fully understand the study.

I therefore freely and knowingly give my informed consent to voluntarily participate in the study. I acknowledge that I have received a copy of this consent form and I expressly authorize, by signing below, the processing of my personal data for the purposes previously described, in connection with the management and implementation of this research project.

In ____________________, on the ______________________

Name and surname of participant/representative:	Name and surname of investigator:
______________________________	______________________________
**Signature**	**Signature**

RIGHT OF WITHDRAWAL

(For participants who wish to exercise their right to withdraw consent)

I, (name of participant/patient or their representative):

- On my own behalf (check if applicable)
- On behalf of another person (check if applicable).

  Name of the person I represent: ________________________________

Acknowledging that I have taken into account their previously expressed wishes or objections regarding this study,

hereby withdraw the informed consent previously granted on the ____ day of ___________, 20, and I no longer wish to continue participating in the study, which I consider concluded as of the above-mentioned date.
I acknowledge that I have received a copy of this withdrawal form.

Name and surname of participant/representative:	Name and surname of investigator:
______________________________	______________________________
**Signature**	**Signature**
